# An Integrated Immunometabolic Signature Predicts Prognosis and Immunotherapy Response in ccRCC and Identifies *UCN*-Mediated Immune Evasion as a Therapeutic Vulnerability: Evidence from In Vitro and In Vivo Studies

**DOI:** 10.3390/cancers18091373

**Published:** 2026-04-25

**Authors:** Zhinan Xia, Yu Dong, Xin Zhang, Wenjiao Xia, Hongru Wang, Yiyang Zhou, Yiming Qi, Yulan Liang, Zhijian Li, Yuhang Zhang, Zhiming Cui, Keliang Wang, Cheng Zhang

**Affiliations:** 1 Department of Urology, The Fourth Affiliated Hospital of Harbin Medical University, Harbin 150000, China; 2 Department of Urology, The Fourth Affiliated Hospital of School of Medicine, and International School of Medicine, International Institutes of Medicine, Zhejiang University, Yiwu 322000, China; 3 Department of Radiology, Second Affiliated Hospital of Naval Medical University, Shanghai 200000, China; 4 Department of Radiology, 920th Hospital of Joint Logistics Support Force, People’s Liberation Army (PLA), Kunming 650000, China; 5 Department of Radiology, The Fourth Affiliated Hospital of School of Medicine, and International School of Medicine, International Institutes of Medicine, Zhejiang University, Yiwu 322000, China

**Keywords:** clear cell renal cell carcinoma, molecular subtype, immunometabolism, *UCN*, immune microenvironment, immunotherapy

## Abstract

Background/Objectives: Clear cell renal cell carcinoma (ccRCC) involves complex interactions between immune evasion and metabolic reprogramming. This study aimed to characterize ccRCC through integrated immunometabolic profiling, develop a prognostic signature, and investigate the functional role of the key driver gene UCN using in vitro and in vivo approaches. Methods: Integrated immunometabolic profiling was performed to identify molecular subtypes and establish a prognostic gene signature. Two distinct molecular subtypes were identified, and a 9-gene Immune Metabolic Index (IMI) was constructed. The functional role of the key driver gene UCN was investigated through in vitro functional assays and in vivo xenograft models in BALB/c mice, including combination with PD-1 blockade. Results: Two molecular subtypes with significant survival differences (*p* < 0.001) were identified. The established IMI demonstrated high prognostic accuracy, with Area Under the Curve (AUC) values of 0.813, 0.751, and 0.779 at 1-, 3-, and 5-year intervals, respectively. UCN was identified as the highest-risk gene in the signature. Functional assays showed that UCN silencing significantly inhibited cell proliferation and migration (*p* < 0.05). In BALB/c mouse xenograft models, UCN silencing remodeled the tumor microenvironment by increasing CD8+ T cell infiltration and reducing regulatory T cells (*p* < 0.01). Furthermore, UCN knockdown significantly suppressed tumor growth and synergized with PD-1 blockade to enhance antitumor efficacy (*p* < 0.001). Conclusions: The IMI is a robust tool for risk stratification in ccRCC. Targeting the UCN-driven immunometabolic axis represents a promising therapeutic strategy to overcome immune resistance in ccRCC.

## Simple Summary

Kidney cancer progression involves complex interactions between the body’s immune system and the energy usage of cancer cells. This study investigates these connections to better understand patient outcomes and identify new treatment strategies. The authors analyzed genetic data to create a scoring system, called the Immune Metabolic Index (IMI), which accurately predicts how patients with kidney cancer may respond to therapy. Importantly, the research identified a specific gene, *UCN*, as a key player in this process. Laboratory experiments revealed that blocking *UCN* significantly slowed cancer cell growth and spread. In animal models, suppressing *UCN* remodeled the tumor environment by increasing cancer-fighting immune cells while reducing suppressive ones. Furthermore, targeting *UCN* enhanced the effectiveness of common immunotherapies, suggesting that combining *UCN* inhibitors with existing treatments could offer a promising new approach for kidney cancer patients.

## 1. Introduction

Clear cell renal cell carcinoma (ccRCC), an insidious malignant neoplasm that afflicts the urological system, is distinguished by its proclivity to originate from proximal renal tubular cells. This particular type of cancer stands out as the most commonly occurring histological subtype of renal cell carcinoma (RCC), accounting for a substantial proportion of all RCC cases, estimated to be around 70% [1]. Globally, approximately 430,000 individuals are diagnosed with RCC annually, with over 175,000 associated fatalities. Moreover, there has been a steady rise in the incidence and mortality rates of RCC over the last few decades, increasing by an estimated 2–4% each year [2]. At the time of diagnosis, nearly 30% of ccRCC patients develop distant metastases, while more than 20% may experience relapse or distant metastasis after undergoing radical nephrectomy [3]. Notably, ccRCC has been observed to be largely resistant to radiotherapy and chemotherapy, which necessitates the use of multitarget tyrosine kinase inhibitors (TKIs) and immune checkpoint inhibitors (ICIs) as the mainstay treatment modalities for metastatic RCC patients [4]. Although the field of immunotherapy has seen remarkable advancements, the clinical responses of ccRCC patients to immunotherapy remain highly variable, with only a limited number of cases exhibiting successful outcomes [5]. Furthermore, while several potential biomarkers are currently under evaluation, the development of validated markers for predicting prognosis and guiding treatment decisions in metastatic ccRCC patients remains an area requiring significant advancement [6]. Therefore, the development of a robust prognostic signature that can more precisely estimate the prognosis of ccRCC patients and aid in the effective implementation of immunotherapy is of utmost importance.

Two pivotal features of tumor biology that exert a considerable impact on the development and advancement of tumors are immune evasion [7] and metabolic reprogramming [8]. The complex interplay between the immune system, which defends against pathogens, and the metabolic system, responsible for energy production, is widely acknowledged. Moreover, the immune system is closely linked with metabolism beyond pathogen defense, and this relationship has critical implications for tumorigenesis [9]. However, it is noteworthy that the dysregulated metabolic activity in tumor cells often leads to metabolic stress on tumor-infiltrating immune cells, resulting in a compromised antitumor immune response [10,11]. The TME is impacted by certain metabolites secreted by tumors, which hinder the ability of CD8+ T cells to eliminate tumor cells [12]. Moreover, oncometabolites have been found to have extracellular roles in the TME that go beyond their established intracellular functions, facilitating intercellular communication [13]. As a result, several prognostic signatures have been developed, based on metabolism or immunity, to aid in predicting the prognosis of ccRCC patients and guiding immunotherapy, yielding promising outcomes [14,15,16,17,18,19,20,21,22]. However, ccRCC is not just a highly metabolic tumor, but also one with high immunogenicity, characterized by substantial immune cell infiltration. This uniqueness, along with its shared characteristics with other tumors, makes the TME of ccRCC distinctive. The above evidence suggests that exploring the prognostic implications of the interaction between immune and metabolic factors is crucial.

In this study, a comprehensive approach was adopted by merging genes related to both immunity and metabolism for molecular classification of ccRCC. Two molecular subtypes showed significant differences in prognosis, TME, function, and gene mutations, and a prognostic signature was developed based on the differentially expressed genes (DEGs) of the two molecular subtypes. A meticulous integrative analysis was conducted to assess its prognostic value on a larger scale. We validated the expression of nine signature genes through qRT-PCR experiments on four cell lines. In addition, a comparison was made between our model and nine other models, including the well-established ClearCode34, as well as eight models that solely use immune or metabolic genes, and it was discovered that our combined model surpassed them all [14,15,16,17,18,19,20,21,22]. Furthermore, a prognostic nomogram was devised, which serves as a quantitative tool to predict the risk of prognosis in patients with ccRCC. Finally, we knocked down the gene *UCN*, which indicated the poorest prognosis in the signature, demonstrating its role in promoting proliferation and migratory invasion in two ccRCC cell lines. In vivo experiments confirmed that *UCN* knockdown suppresses ccRCC tumor growth and reshapes the immune microenvironment by increasing CD8+ T cell infiltration and reducing regulatory T cells (Tregs), with enhanced anti-tumor effects when combined with PD-1 inhibition. Our aim is to provide a theoretical and practical basis for precise stratification management and novel therapeutic targets in ccRCC immunotherapy.

## 2. Materials and Methods

### 2.1. Data Acquisition and Identification of Immune- and Metabolism-Related Genes (IMRGs)

The clinical data and RNA-seq profiles of ccRCC samples could be attained from the Cancer Genome Atlas (TCGA) database (https://portal.gdc.cancer.gov/, accessed on 1 January 2024). To divide TCGA-KIRC participants into training and testing cohorts, a ratio of 7:3 was utilized, resulting in a training cohort of *n* = 371 and a testing cohort of *n* = 159. The genes associated with immunity for this study were obtained from the ImmPort database (https://www.immport.org, accessed on 1 January 2024), whereas genes associated with metabolism were attained via downloading the file named “c2.cp.kegg. v7.4. symbols” from MSigDB 7.4.

Transcriptional and clinical profiles of the E-MTAB-1980 project from the Array Express database were employed as an external validation dataset [23]. IMvigor210, an external cohort comprising 348 patients treated with anti-PD-1 therapy, was downloaded for subsequent immunotherapy response validation [24]. We obtained immunohistochemical images of ccRCC from the publicly accessible Human Protein Atlas (HPA) database [25].

### 2.2. An Exploration of a Clustering Algorithm Utilizing Non-Negative Matrix Factorization (NMF)

To analyze the differentially expressed IMRGs in ccRCC and normal samples, we applied the Wilcoxon signed-rank test with log2 [fold-change (FC)] > 2 and false discovery rate (FDR) < 0.05 as the set criteria. Following a univariate Cox analysis, an attempt was made to cluster ccRCC samples via the NMF method. Specifically, we opted for the “nsNMF” algorithm and performed 100 iterations while setting the number of clusters K within the range of 2 to 10 [26].

### 2.3. Analysis of TME and Cellular Components

To determine the ratio of immune/stromal components and tumor purity in the tumor microenvironment for each molecular subtype, an attempt was made to utilize the “ESTIMATE” algorithm [27]. Furthermore, the “xCell” and “TIMER” algorithms were selected for immune microenvironment quantification [28,29]. An immune modulator-related gene list was obtained from the GeneCards database and subsequently filtered by functional relevance score [30]. We conducted a single-sample gene set enrichment analysis (ssGSEA) utilizing the R package “gsva” [31] to assess the enrichment scores of immunocytes and immune function-relevant pathways. The relationships among the IMI score, immune cell fractions, and immune functions were examined via a Spearman correlation analysis. Furthermore, a correlation analysis of IMRGs expression levels with immune functions was also performed.

### 2.4. Genomic Alterations Analysis

The patient variants file was obtained from the TCGA repository. Utilizing the “maftools” package (all R packages used in this study were obtained from Bioconductor [https://bioconductor.org/packages/; accessed on 1 January 2024]), we assessed mutated genes across various IMI categories and calculated the tumor mutation burden (TMB) of each individual [32]. Additionally, this software was also harnessed to generate an oncoplot depicting the variant landscape. Furthermore, the “maftools” package was utilized to produce a heatmap displaying co-occurring variants, unrelated variants, and mutually exclusive variants.

### 2.5. Development of a Prognostic Signature Utilizing Clusters Differentially Expressed IMRGs

Univariate Cox regression analysis was utilized to identify prognosis-associated IMRGs that differentially expressed across clusters. The LASSO Cox regression analysis was then undertaken via the “glmnet” R package for the purpose of developing the prognostic signature. The IMI was calculated in the following manner:

IMI = ∑i Coef (Gene i) × Exp (Gene i)

(“Coef” parameter represents the non-zero regression coefficients that were calculated using univariate Cox regression analysis. Furthermore, the “Exp” parameter denotes the expression values of the genes derived from the prognostic risk score model) [26].

### 2.6. Survival Analysis and Clinical Correlation Analysis

The illustration of the survival curve was undertaken via the Kaplan–Meier plotter, while the correlation between clinicopathological features in the high- and low-risk groups was visualized using the “ggplot2” package. It is noteworthy that *p* < 0.05 was indicative of statistical significance, reflecting a high level of confidence in the validity of our results [26].

### 2.7. Construction and Validation of the Prognostic Nomogram

In this study, a nomogram was developed that incorporates several prognostic variables, including the IMRGs prognostic signature and clinical features, to predict survival probabilities for ccRCC patients over 1-, 3-, and 5-year periods. The construction of this tool involved a multi-step process that included identifying prognostic variables through statistical analysis, developing a predictive model, and creating a user-friendly visualization of the model using graphical representation. To figure out the precision and efficacy of the nomogram in predicting outcomes, ROC and calibration curves were generated using the “timeROC” and “rms” R packages. These curves allow for an in-depth assessment of the robustness and reliability of our approach [26].

### 2.8. Evaluation of the Response to Immunotherapy

The calculation of the Immunophenoscore (IPS) involved using four primary factors, specifically MHC molecules, immunomodulators, effector cells, and suppressor cells [33]. These factors were obtained from the TCIA database (https://tcia.at/home, accessed on 1 January 2024). The IPS was utilized to predict the therapeutic response to PD-1 and CTLA-4, which are the two most important immune checkpoints [33].

### 2.9. Antineoplastic Drug Sensitivity Prediction

The difference in the half-maximal inhibitory concentration (IC50) between the groups was assessed using the Wilcoxon signed-rank test. The outcomes were then visually represented as a box plot with the aid of “pRRophetic” and “ggplot2” [34].

### 2.10. Cell Culture

Human ccRCC cell line 786-O (Accession Number: CVCL_1051) and mouse ccRCC cell line Renca (CVCL_2174) were obtained from American Type Culture Collection (ATCC) (Manassas, Virginia) and cultured in RPMI 1640 medium (Procell, Wuhan, China) containing 10% fetal bovine serum (Procell, China) and Penicillin–Streptomycin (Procell, China). Human renal tubular epithelial cell line HK2 (CVCL_YE28), ccRCC cell line A498 (CVCL_1056), and ACHN (CVCL_1067) were obtained from the National Collection of Authenticated Cell Cultures (Shanghai, China) and cultured in the EMEM medium (Procell, China) containing 10% fetal bovine serum (Procell, China) and Penicillin–Streptomycin (Procell, China).

### 2.11. Quantitative Real-Time Polymerase Chain Reaction (qRT-PCR)

Total RNA from cells and tissues was extracted utilizing RNAiso Plus (TaKaRa, Kusatsu, Japan). Reverse transcription kits (TaKaRa, Japan) and PCR kits (SYBR Green) (TaKaRa, Japan) were used to perform qRT-PCR according to the manufacturer’s instructions. The primer sequences of primers are provided in Supplementary Table S5. The results were analyzed by the 2^−ΔΔCt^ method to quantify fold changes. The molecular biology experimental methods described below were adapted from our previously published article, with minor modifications [35].

### 2.12. RNA Interference

The siRNAs for *UCN* were purchased from GenePharma (Suzhou, China), and their sequences are shown in Supplementary Table S6. Transfection of the siRNA was performed using jetPRIME (PolyPlus, Suzhou, China). Specific shRNA sequences were delivered to target cells using a lentiviral vector system. Following puromycin selection, quantitative real-time PCR was performed to validate knockdown efficiency [35].

### 2.13. Cell Counting Kit-8 (CCK-8) Assay

Cell proliferation was detected via a CCK-8 assay (Beyotime, Shanghai, China). Transfected cells were plated into 96-well plates (2000 cells/well) for 12 h. After different time periods (0, 24, 48, and 72 h), the culture medium was mixed with 10% CCK-8 and added to each well for 2 h. The absorbance in each well was measured at 450 nm with a spectrophotometer to obtain the optical density (OD) value [35].

### 2.14. Wound-Healing Assay

A wound-healing assay was performed to evaluate the migration ability of cells. Cells (2 × 10^6^ cells/well) were cultured in six-well plates with coverslips for 16 h (786-O) or 24 h (ACHN). A straight scratch was made with a sterile pipette tip, and the damaged cells were washed off. The wound-healing areas were observed under a microscope at 0 and 16 h (786-O) or 24 h (ACHN) [35].

### 2.15. Trans-Well Invasion Assay

Trans-well assays were performed to evaluate the invasion ability of cells. 786-O cells (1 × 10^4^ cells/well) and ACHN cells (2 × 10^4^ cells/well) were placed into the upper Trans-well cell culture chambers (8 μm pore size) coated with Matrigel (Corning, NY, USA), and the lower chambers were filled with medium supplemented with 20% FBS. After 24 h, the invasive cells were fixed with 4% paraformaldehyde for 30 min and then stained with 0.5% crystal violet for 20 min [35].

### 2.16. Western Blot Assay

For Western blotting, proteins were extracted by lysing cultured cells in a RIPA buffer containing protease inhibitors and quantified using a BCA assay kit (Beyotime, Shanghai, China). Later, heat-denatured proteins mixed with 1 × loading buffer were separated via SDS–PAGE and then transferred onto PVDF membranes (Millipore, Burlington, MA, USA). Finally, the blots were exposed to enhanced chemiluminescence reagents using ImageQuant™ LAS 4000 after incubation with the UCN antibodies (PA5-75189, Invitrogen, Carlsbad, CA, USA), followed by an HRP-conjugated secondary antibody (31460, Invitrogen, USA) [35].

### 2.17. Establishment and Intervention of Subcutaneous Tumor Model in Mice

For in vivo experiments, healthy male Balb/C mice (6–8 weeks old, body weight ~20 g) purchased from Charles River Laboratories (Beijing, China) were randomly assigned into four groups (*n* = 5 per group). A total of 1 × 10^6^ Renca NC or sh *UCN*-treated cells suspended in sterile PBS were injected subcutaneously at a single site on the right dorsal flank (one site per mouse). Tumor length and width were measured daily with calipers to monitor growth. When any tumor reached a volume of 100 mm^3^ (designated as day 0), mice received intraperitoneal injections of either an in vivo mAb anti-mouse PD-1 (CD279) (BE0033-2, BioXCell, Lebanon, NH, USA) or an IgG2a isotype control (BE0091, BioXCell, USA) (200 µg per mouse) every three days. On day 30, euthanasia was performed using cervical dislocation, carried out rapidly by trained personnel. Death was confirmed by cessation of respiration and heartbeat and loss of the righting reflex, after which tumors were harvested, their volumes and weights measured, and samples processed for immunological analyses [35].

### 2.18. Flow Cytometry Analysis

For flow cytometry, freshly excised tumors were minced and digested in a solution containing collagenase IV and DNase I to prepare single-cell suspensions. The cell suspensions were filtered to remove debris, and immune cells were enriched with density gradient centrifugation. Sorted cells were incubated with stimulants to induce functional molecule expression, followed by Fc receptor blocking (553141, BD, Franklin Lakes, NJ, USA) to minimize non-specific binding. Surface staining was performed with fluorescence-conjugated antibodies. After fixation and permeabilization, intracellular staining was conducted using antibodies against *Foxp3* (12-5773-82, Thermo, Waltham, MA, USA). Data were acquired using a flow cytometer, and sequential gating was used to identify CD45+ leukocytes (557659, BD, USA), CD3+ T cells (561798, BD, USA), CD8+ T cells (563152, BD, USA), Tregs and to assess the proportion of PD-1 (566514, BD, USA) positive cells within the CD8+ T cell population [35].

### 2.19. Histology

Harvested mouse tissues were fixed in 4% paraformaldehyde (Beyotime, Shanghai, China) overnight, embedded in paraffin, and sectioned using a microtome. Tissue sections were stained with H&E using standard protocols. For IHC analysis, sections were boiled in antigen retrieval solution for 15 min and then brought to room temperature. Subsequently, sections were incubated with specific antibodies, and using the Biotin–Streptomycin Complex System (Zhongshan Golden Bridge, Beijing, China) or a multiplex fluorescent immunohistochemical staining kit (Absin, Shanghai, China) according to the manufacturer’s instructions to visualize the antigen. Images were taken using Digital Slide Scanners [35].

### 2.20. Statistical Analysis

All data analyses were performed using R software (version 4.3.2). Comparisons between two groups were conducted using *t*-tests or non-parametric tests, and comparisons among multiple groups were conducted using analysis of variance. Kaplan–Meier method and Log-rank test were used for survival curve comparison. Correlation analyses were performed using Spearman correlation coefficients. All statistical tests were two-sided, and *p* < 0.05 was considered statistically significant.

## 3. Results

### 3.1. Identification of Differentially Expressed IMRGs and ccRCC Molecular Subtypes

**Figure 1:**
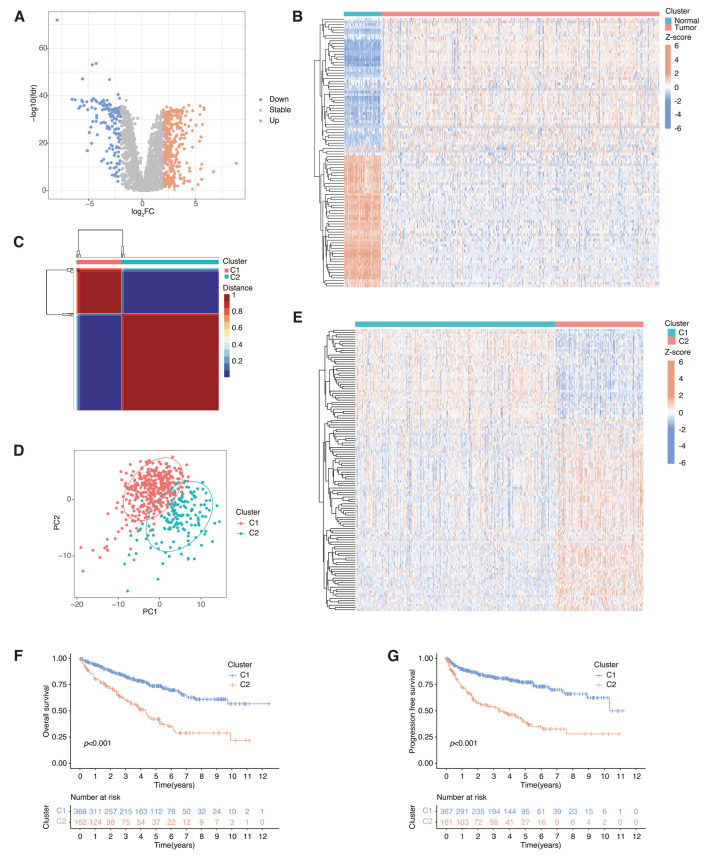
The process of identifying differentially expressed IMRGs and molecular subtypes in ccRCC: (**A**) IMRGs that were differentially expressed were denoted by red dots for upregulation and blue dots for downregulation. (**B**) Heatmaps were used to visually represent the top differentially expressed genes. (**C**) A heatmap of the nsNMF consensus matrix was generated to classify ccRCC into two molecular subtypes. (**D**) A PCA plot was applied to show significant differences between clusters. (**E**) The gene expression heatmap shows how the identified IMRGs were expressed across the two molecular subtypes. (**F**,**G**) In order to make a comparison between the two molecular subtypes, the researcher employed the Kaplan–Meier curve to assess and contrast the OS and PFS.

The TCGA-KIRC database was utilized to obtain transcriptome and clinical information of 539 ccRCC samples and 72 normal kidney samples. The quantification of gene expression levels pertaining to immune function (as elucidated in Supplementary Table S1) and metabolic processes (as explicated in Supplementary Table S2) has been meticulously extracted. After carrying out the Wilcoxon signed-rank test, a comparative analysis was conducted between tumor samples and normal samples. Based on the criteria of |log2FC| > 2 and FDR < 0.05 (Figure 1A,B), following rigorous analysis, a comprehensive set of 519 differentially expressed immune- and metabolism-related genes (IMRGs), including 374 overexpressed and 145 under-expressed genes (as listed in Supplementary Table S3), were successfully identified. Furthermore, employing univariate Cox regression, a subset of 125 IMRGs that were deemed to be prognostically relevant for the studied condition was also detected (detailed in Supplementary Table S4).

Subsequently, the NMF clustering algorithm was applied to the prognosis-related IMRGs, resulting in the identification of two molecular subtypes (Figure 1C). The indicators of cophenetic, silhouette, and dispersion were mainly used to determine the optimal rank value (Supplementary Figure S1). According to the clustering effect shown in the PCA plot, we can confirm significant differences among the molecular subgroups we have distinguished (Figure 1D). The heatmap demonstrated the expression of the prognosis-related IMRGs in both molecular subtypes (Figure 1E). On the basis of the statistical analysis, the survival outcomes in terms of overall survival (OS) and progression-free survival (PFS) were significantly superior for cluster 1 when compared to cluster 2, as illustrated by Figure 1F,G.

### 3.2. Functional Enrichment Analysis of Clusters

**Figure 2:**
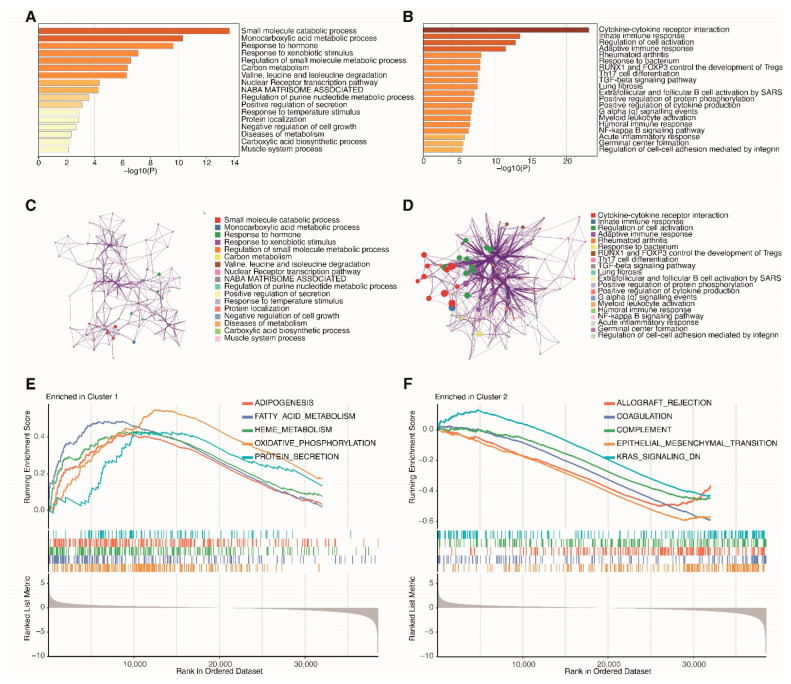
Functional annotation of DEGs between the two molecular subtypes. (**A**,**B**) Metascape bar chart displays the top 20 non-redundant significantly enriched pathways in Cluster 1 and Cluster 2, respectively. (**C**,**D**) Metascape enrichment networks display both intra-pathway and inter-pathway similarities among enriched terms within Cluster 1 and Cluster 2, respectively, with color coding reflecting cluster annotations. (**E**) GSEA based on hallmark gene sets reveals the pathways enriched in Cluster 1. (**F**) GSEA based on hallmark gene sets reveals the pathways enriched in Cluster 2.

To elucidate potential discrepancies in biological functions between different clusters, functional enrichment analyses based on the DEGs were introduced. Our analysis unveiled significant enrichments in several immune-related GO terms and pathways within the Cluster 2 group. These enrichments included terms related to the innate immune response, adaptive immune response, humoral immune response, and acute inflammatory response (Figure 2B). Conversely, metabolism-related GO terms and pathways were notably enriched in the Cluster 1 group. These encompassed processes like small molecule catabolic processes, monocarboxylic acid metabolic processes, regulation of small molecule metabolic processes, and more (Figure 2A). Metascape enrichment networks display both intra-pathway and inter-pathway similarities (Figure 2C,D). Furthermore, the Hallmark gene set-based GSEA results highlighted a pronounced association of Cluster 1 with metabolism (Figure 2E), while the association between Cluster 2 and metabolism was not significant (Figure 2F). These findings suggest that the two clusters may have distinct biological characteristics that could influence their prognosis and response to therapy.

### 3.3. Comparison of Tumor Immune Microenvironment Between the Clusters

**Figure 3:**
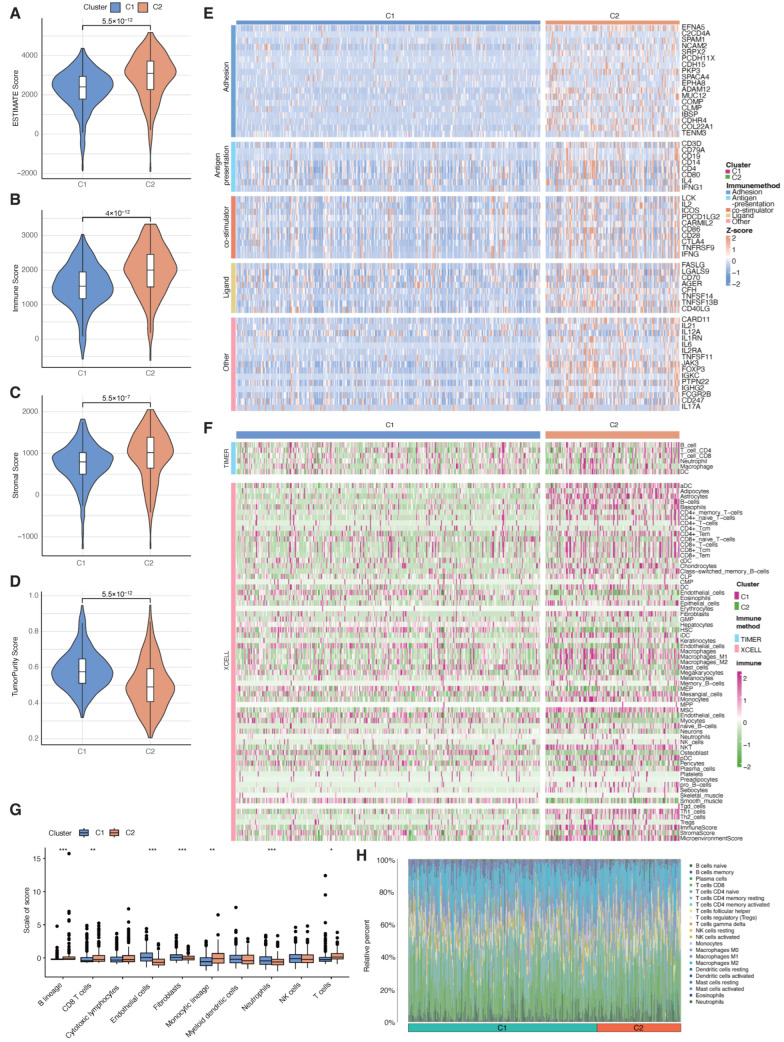
Comparative analysis of the distinct tumor immune microenvironment between the two molecular subtypes. (**A**–**D**) An analysis of the immune microenvironment using the ESTIMATE algorithm revealed significant differences between the two clusters. (**E**) Heatmap showcasing the landscape of immune-related functions in the two clusters. (**F**) Heatmap illustrating the landscape of immune cell infiltration in the two clusters. (**G**) The cellular composition analysis conducted using the MCP counter algorithm demonstrated significant variations in the proportions of different cell types between the two subtypes. An attempt was made to denote statistical significance by * *p* < 0.05, ** *p* < 0.01, and *** *p* < 0.001. (**H**) The bar plot showed the proportion of 22 types of tumor-infiltrating immune cells in the two clusters.

The aforementioned result from GO functional enrichment analysis demonstrated a pronounced enrichment of immune pathways in Cluster 2. To delve deeper into this observation, ESTIMATE algorithm was conducted to evaluate stromal scores, ESTIMATE scores, immune scores, and tumor purity of the ccRCC patients. The outcomes indicated a substantial difference, with Cluster 2 patients exhibiting significantly higher ESTIMATE scores (*p* < 0.0001), stromal score, and immune scores (*p* < 0.0001), compared with the Cluster 1 patients (Figure 3A–C). Furthermore, tumor purity was notably lower in Cluster 2 (*p* < 0.0001, Figure 3D). These findings collectively suggested a substantial activation of the immune state in Cluster 2.

Next, we employed a variety of algorithms to comprehensively evaluate the tumor immune microenvironment. We assessed the expression of various immunomodulators between the clusters, including adhesion molecules, antigen presentation factors, co-stimulatory molecules, ligands, and other immunomodulatory markers. These molecules were upregulated in Cluster 2 (Figure 3E). Furthermore, we noticed a markedly elevated infiltration of immunocytes in Cluster 2 (Figure 3B). This was reflected in a greater abundance of B cells, CD8+ T cells, CD4+ T cells, and Macrophages (Figure 3F). A barplot was utilized to provide the immune cell landscapes across all ccRCC patients (Figure 3H). For further validation, ssGSEA was applied to evaluate the enrichment scores for immunocytes (Figure 3G). These results provided additional confirmation of our previous discoveries, indicating that the ccRCC patients from Cluster 2 not only exhibited increased immune cell infiltration but also demonstrated heightened immune activation.

### 3.4. Comparison of Genomic Alterations Between the Clusters

**Figure 4:**
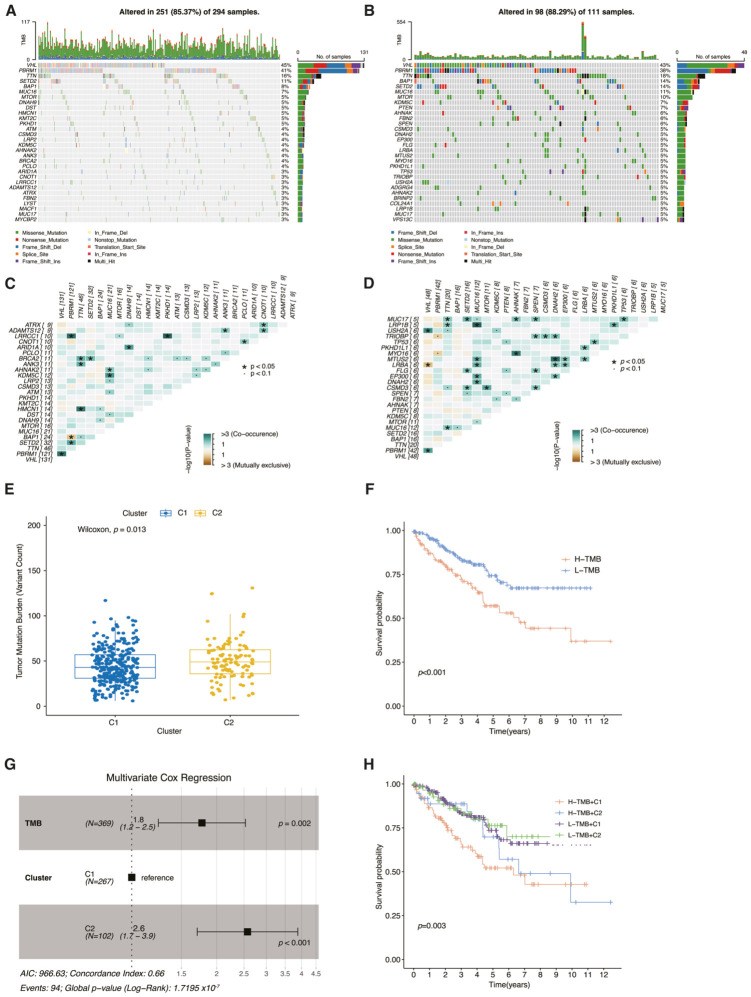
Comparison of genomic alteration landscapes between the two molecular subtypes. (**A**) Oncoplot demonstrated the 30 most frequently mutated genes in Cluster 1. (**B**) Oncoplot demonstrated the 30 most frequently mutated genes in Cluster 2. (**C**) Heatmap illustrating the co-mutated states of the commonly mutated genes in Cluster 1. (**D**) Heatmap illustrating the co-mutated states of the commonly mutated genes in Cluster 2. (**E**) The boxplot illustrates the distinct tumor mutation frequencies between Cluster 1 and Cluster 2. (**F**) The Kaplan–Meier curve shows the overall survival rates of patients with high and low tumor mutation burdens. (**G**) Multivariate Cox regression analysis of tumor mutation burden (TMB) and immunometabolic clusters. (**H**) Kaplan–Meier survival curves for ccRCC patients stratified by both TMB status (high vs. low) and immunometabolic clusters (C1 vs. C2).

By integrating the mutation data of each patient, we conducted genomic mutation analysis for the clusters. It was observed that in both clusters, missense mutations were the predominant variant classification (Supplementary Figure S2A,G). Furthermore, among the variant types, single-nucleotide variants (SNVs) were the most prevalent, with C > T mutations being the primary SNV subtype (Supplementary Figure S2B,C,H,I). Overall, patients in Group 2 exhibited a higher mutation frequency and a greater median number of variants (Figure 4A,B, Supplementary Figure S2D,J). Notably, in both clusters, the ccRCC-triggering gene VHL was found to co-occur with PBRM1, which is an important co-driver in ccRCC tumorigenesis (Figure 4C,D) [36]. Genomic alterations serve as crucial indicators of genomic instability. Our study findings demonstrate that Cluster2 exhibited a significantly higher tumor mutation burden (TMB) compared to Cluster1 (Figure 4E). Additionally, ccRCC patients with a high TMB experience a poorer prognosis (Figure 4F). To further investigate whether TMB acts as an independent prognostic factor or if its impact is primarily driven by its association with the Cluster 2 phenotype, we performed a multivariate Cox proportional hazards analysis. The results revealed that high TMB remained a statistically significant independent risk factor for poor survival after adjusting for the cluster subtype (Figure 4G). Furthermore, stratified survival analysis demonstrated that high TMB consistently correlated with worse clinical outcomes within both the Cluster 1 and Cluster 2 subgroups (Figure 4H). These findings collectively indicate that the prognostic value of TMB is independent of the immunometabolic clustering, reinforcing its role as a distinct driver of poor prognosis in ccRCC.

### 3.5. Construction of a Prognostic Signature Based on DEGs Between the Clusters

**Figure 5:**
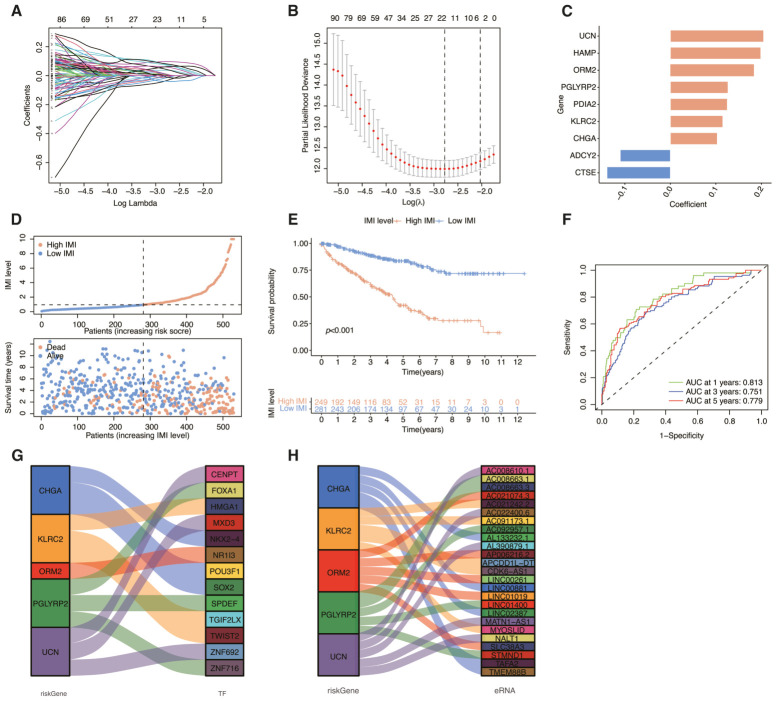
Constructing a prognostic signature with nine core genes identified from IMRGs that are differentially expressed across clusters. (**A**) Utilizing LASSO regression, the independent variables’ coefficients were calculated to determine their impact on the outcome. (**B**) The logarithmic value for lambda that yielded the most favorable results was pinpointed by the initial black dotted line from the left. (**C**) The bar chart displays genes and coefficients, where coefficients greater than zero are indicated by yellow and coefficients less than zero by blue. (**D**) The scatter plot was utilized to depict the distribution of IMI and survival status. (**E**) The illustration displays a Kaplan–Meier curve representing both high and low IMI groups across the TCGA-KIRC cohort. (**F**) ROC curves depicting the prognostic signature of IMRGs at 1-, 3-, and 5-year intervals are shown in the presentation. (**G**) A Sankey diagram illustrates the potential regulatory relationships between IMRGs and TFs. (**H**) A Sankey diagram illustrates the potential regulatory associations between IMRGs and enhancer-derived RNAs.

To further guide clinical diagnosis and prognostic prediction, we constructed a prognostic prediction model based on molecular subtyping. The TCGA-KIRC cohort underwent a random division into two groups with a ratio of 7:3, which were referred to as the training cohort and test cohort. Leveraging differentially expressed IMRGs between clusters, the TCGA training cohort was employed to develop a prognostic prediction model. The construction of this model involved implementing LASSO Cox regression analysis, which allowed for the identification of key predictors and the development of an accurate prognostic model. Figure 5A,B presents a comprehensive visualization of the coefficients attributed to each independent variable in the LASSO regression, along with the optimal logarithmic value of lambda. An attempt was made to develop a prognostic signature comprising nine genes, including CTSE, KLRC2, PDIA2, HAMP, PGLYRP2, ORM2, CHGA, *UCN*, which are immune-related genes, and ADCY2, a metabolism-related gene. By applying multivariate Cox regression analysis, we calculated an immune-metabolism index (IMI), which serves as a comprehensive indicator of the immune and metabolic status. In order to obtain the required score, an attempt was made to employ a method that involved multiplication of the expression levels of each gene by their corresponding coefficients (Figure 5C):
IMI = −0.139435 × *CTSE* + 0.115443 × *KLRC2* + 0.125361 × *PDIA2* + 0.198961 × *HAMP* + 0.126838 × *PGLYRP2* + 0.183848 × *ORM2* + 0.103308 × *CHGA* + 0.205241 × *UCN* + −0.110254 × *ADCY2*


**Figure 6:**
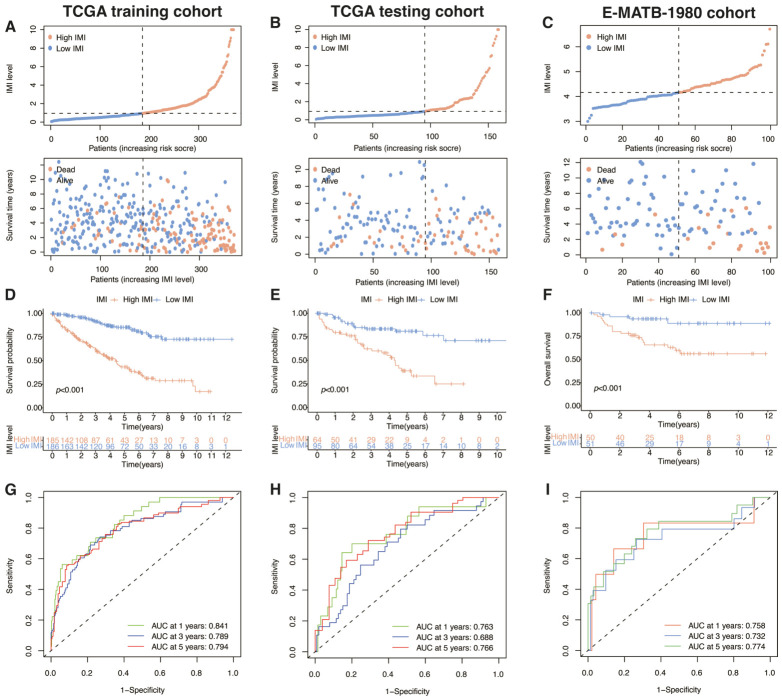
Assessment and confirmation of the predictive performance of the signature in ccRCC. (**A**–**C**) Scatter plots illustrating the survival status and IMI scores of ccRCC patients in the TCGA training group (**A**), the TCGA testing group (**B**), and the E-MATB-1980 external validation group (**C**). (**D**–**F**) Kaplan–Meier curves displaying the overall survival situation per IMI scores of the high-IMI group and low-IMI group in the TCGA training group (**D**), the TCGA testing group (**E**), and the E-MATB-1980 external validation group (**F**). (**G**–**I**) ROC curves demonstrating the predictive performance of IMI with AUC values for 1-year, 3-year, and 5-year OS in ccRCC patients from the TCGA training group (**G**), the TCGA testing group (**H**), and the E-MATB-1980 external validation group (**I**).

By utilizing the median of IMI, patients from the TCGA training cohort were partitioned into two groups based on their level of risk to investigate the correlation between IMI and prognosis. The risk curve and scatter plot illustrated the distribution of IMI and its association with survival outcomes, providing insights into the potential clinical applications of IMI in cancer prognosis (Figure 5D). Furthermore, results from survival analysis displayed that patients categorized as high-IMI had considerably worse prognosis compared to those in the low-IMI group (Figure 5E). In Figure 5F, it can be observed that the prognostic model’s AUCs were evaluated in the entire TCGA-KIRC cohort, with scores of 0.813, 0.751, and 0.779 for 1-, 3-, and 5-year, respectively. Meanwhile, we also explored the potential regulatory elements of these IMRGs, including transcription factors and eRNAs, to investigate the possible mechanisms underlying their expression regulation (Figure 5G,H). Figure 6 showed the results, including the training and testing cohorts mentioned above, as well as another cohort from the Array Express database (E-MTAB-1980). The E-MTAB-1980 dataset was specifically selected as an external validation cohort due to its high-quality transcriptomic profiling, comprehensive long-term clinical follow-up, and its representation of a distinct geographic population, which ensures the cross-population robustness and clinical applicability of our prognostic model. The dataset E-MTAB-1980 contained sequencing and clinical information of 101 ccRCC patients as external validation of the signature. Both the risk curves (Figure 6A–C), survival curves (Figure 6D–F), and AUCs (Figure 6G–I) of the training cohort and two validation cohorts demonstrated the commendable prognostic value of this immunometabolism signature.

### 3.6. Subgroup Analysis Revealed Significant Prognostic Differences Between Groups with High and Low IMI Levels

**Figure 7:**
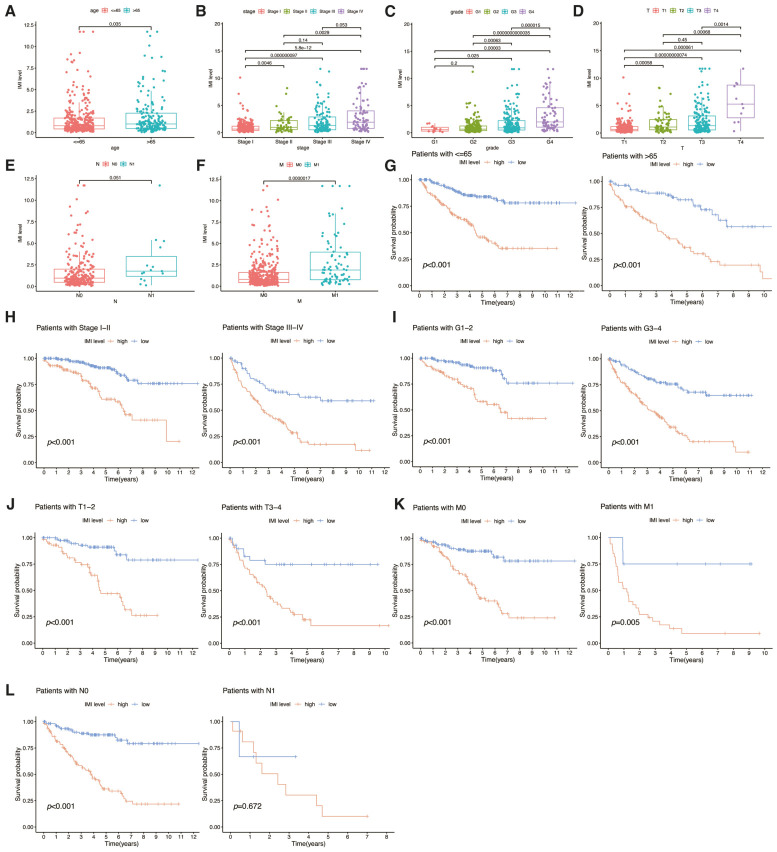
Correlation between clinical features and the prognostic signature. The differences in IMI between various clinical groups, such as age, pathological stage, Fuhrman grade, tumor size, lymph node metastases, and distant metastases, are displayed in (**A**–**F**), respectively. Additionally, survival analysis was conducted on high IMI and low IMI groups for different clinical groups, with the same order as above in (**G**–**L**).

To determine whether the prognostic signature was associated with clinical features, an attempt was made to employ independent *t*-tests for the purpose of evaluating the differences in IMI in the TCGA-KIRC cohort. As the level of age (Figure 7A), pathological stage (Figure 7B), Fuhrman grade (Figure 7C), tumor size (Figure 7D), lymph node metastases (Figure 7E), and distant metastases (Figure 7F) increased, so did IMI. Irrespective of the clinical features—such as age (Figure 7G), pathological stage (Figure 7H), Fuhrman grade (Figure 7I), and TNM, as described above (Figure 7J–L)—significant prognosis-based differences between high and low IMI groups were identified through subgroup analysis. However, in patients with lymph node metastasis, the absence of a significant difference between the high and low IMI groups could be attributed to the limited sample size. These results demonstrate that the prognostic signature has robust prognostic predictive capabilities across different clinical characteristics.

### 3.7. Developing a Nomogram Based on the Prognostic Signature and Assessing Its Clinical Significance

**Figure 8:**
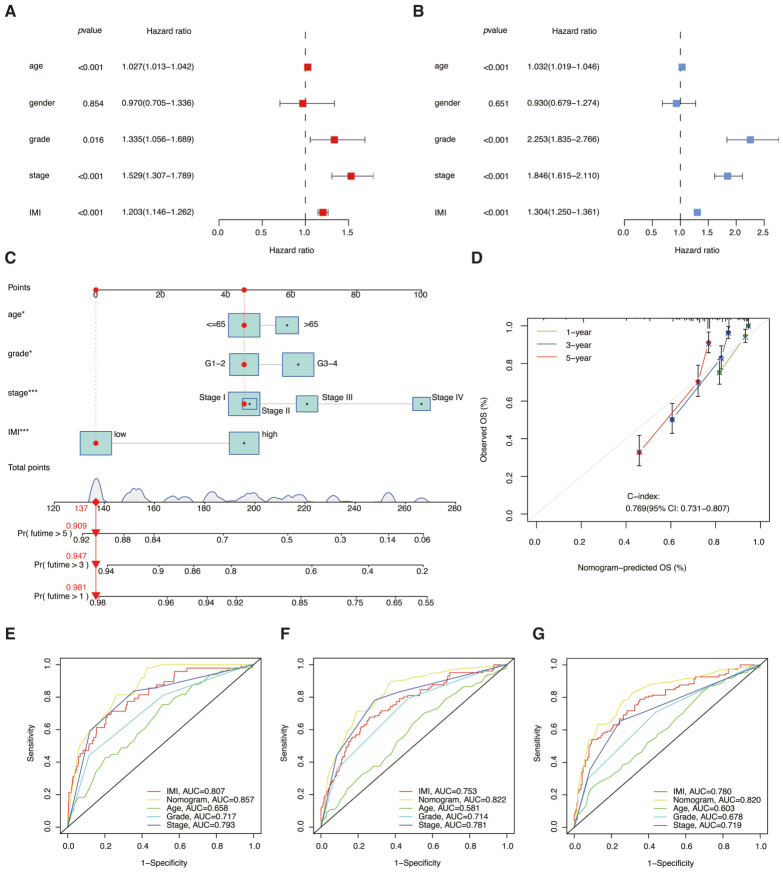
Development of a nomogram utilizing the prognostic signature and examination of its clinical relevance. (**A**,**B**) Univariate [with 95% confidence interval (CI), hazard ratio = 1.304 (1.250–1.361), *p* < 0.001] and multivariate [with 95% CI, hazard ratio = 1.203 (1.146–1.262), *p* < 0.001] Cox regression analyses were performed to assess the prognostic significance of IMI and various clinical features. (**C**) A nomogram was developed to predict the overall survival (OS) at 1-, 3-, and 5-year intervals. The red line was an example of how the nomogram was calculated. * *p* < 0.05; *** *p* < 0.001 (**D**) Calibration curves were plotted to evaluate the accuracy of the nomogram for predicting OS at different time points. (**E**–**G**) ROC curves were employed to evaluate and compare the predictive accuracy of the nomograms along with other variables for survival rates (e.g., 1-, 3-, and 5-year).

To figure out the applicability of the prognostic signature for clinical purposes, an attempt was made to conduct a Cox regression analysis on the complete TCGA-KIRC cohort to assess its independence. The univariate and multivariate regression analyses demonstrated significant correlations between IMI and prognosis, underscoring the potential value of IMI as a prognostic marker for predicting clinical outcomes, as illustrated in Figure 8A,B. It was also observed that age, stage, and grade could serve as independent prognostic factors, along with gender. Therefore, gender was not included in the subsequent construction of the nomogram.

A predictive tool in the form of a nomogram was constructed to estimate the likelihood of survival risk, which is depicted in Figure 8C. To calculate the total score for each patient, the points assigned to each variable on the point scale were added together. On the basis of total points calculated in the previous step using the bottom scale, the likelihood of survival at 1-, 3-, or 5-year intervals was predicted. To ascertain the agreement between the survival predicted by the nomogram and the actual survival, calibration curves were employed (Figure 8D). In addition, the AUCs for the nomogram were found to be higher than those for the separate IMI and other clinical variables (Figure 8E–G). In light of the findings, it can be concluded that the nomogram developed using the prognostic signature was effective in predicting disease progression and had a substantial association with prognosis.

### 3.8. An Evaluation of the Prognostic Signature Through a Comparative Analysis with Signatures Published Beforehand

**Figure 9:**
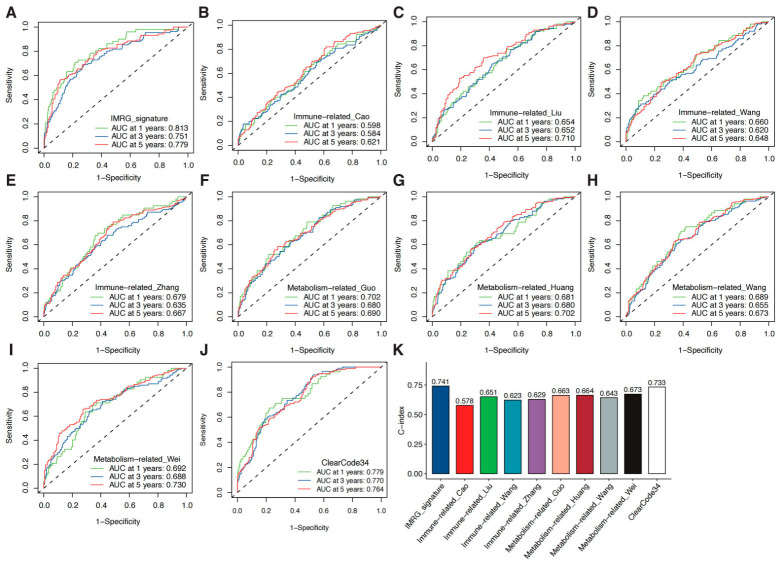
Evaluating our prognostic signature versus other published signatures. (**A**) ROC curves depicting the prognostic signature. (**B**–**J**) The performance of our signature was compared to others by analyzing their ROC curves. (**K**) The prognostic ability of ten signatures was evaluated by comparing their C-indexes.

To investigate the predictive performance of the model associated with the immune system and metabolism, we compared it with a well-established prognostic signature, ClearCode34 [14], as well as eight prognostic signatures published within the last five years that were solely immune-related or metabolism-related. These eight signatures included four immune-related prognostic models, namely Immune-related_Cao, Immune-related_Liu, Immune-related_Wang, and Immune-related_Zhang, as well as four metabolism-related prognostic models, involving Metabolism-related_Guo, Metabolism-related_Huang, Metabolism-related_Wang, and Metabolism-related_Wei [15,16,17,18,19,20,21,22]. The ability to classify ccRCC samples into good and poor prognosis groups was observed in all ten published signatures, with statistically significant differences noted in outcomes for both groups (Supplementary Figure S3A–C). However, the ROC curve analysis revealed that the AUCs of our model were superior to those of the aforementioned published signatures (Figure 9A–J). Additionally, our model achieved the highest C-index value of 0.741, whereas the other models exhibited C-index values ranging from 0.578 to 0.733 (Figure 9K). Of note, the ClearCode34 signature demonstrated predictive performance close to that of our model; however, ClearCode34 utilized 34 genes, whereas our model compressed this to only 9 genes. The other eight published signatures also employed fewer than 10 genes, but their performance was significantly inferior to ours. These findings consistently demonstrate the superior performance of our proposed prognostic signature.

### 3.9. Assessing the Predictive Value of Prognostic Signature in Immune Landscape and Immunotherapy Response

**Figure 10:**
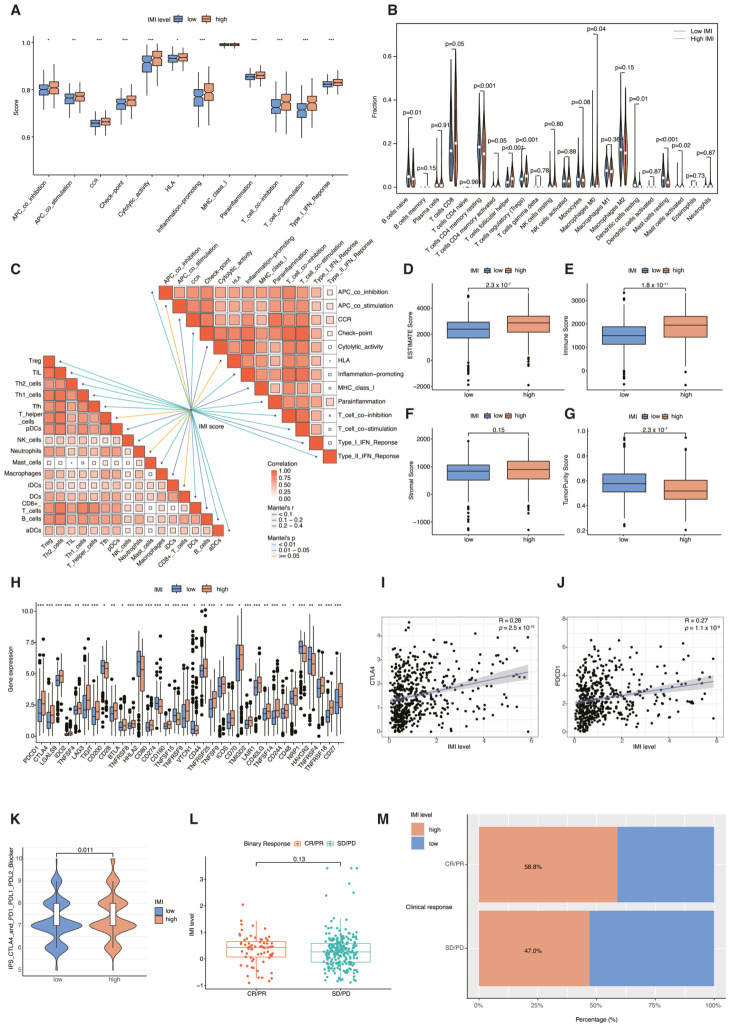
Comparison of immune landscape and immunotherapy response between low- and high-IMI groups. (**A**) ssGSEA analysis for the disparities in immune-related functions between the low- and high-IMI groups. (**B**) Analysis of the fraction of infiltrating immune cells between the two groups. (**C**) Butterfly plot illustrating the association between IMI and the fraction of immune cells and immune-related functions. (**D**–**G**) An analysis of the immune microenvironment using the ESTIMATE algorithm revealed significant differences between the two IMI groups. (**H**) Boxplot illustrating the disparity in expression levels of immune checkpoint genes between the low-IMI and high-IMI groups. * *p* < 0.05; ** *p* < 0.01; *** *p* < 0.001. (**I**) Pearson correlation analysis revealing the association between IMI and *CTLA4* expression. (**J**) Pearson correlation analysis revealing the association between IMI and *PDCD1* expression. (**K**) Violin plot depicting the difference in response to ICIs between the low- and high-IMI groups using the IPS algorithm. (**L**) Boxplot displaying the distribution of IMI scores among patients with different immunotherapy responses. (**M**) Barplot displaying the percentage of responses to immunotherapy in the low- and high-IMI groups.

To evaluate the enrichment scores of immune cells and the activity of immune-related functions, the ssGSEA algorithm was applied. Results revealed that patients in the high-IMI group tended towards an immune-activated state, reflected in elevated levels of various immune-related functions (such as immune checkpoints, T cell co-stimulation, CCR, inflammation-promotion) (Figure 10A), as well as higher proportions of immune cells (CD8+ T cells, follicular helper T cells, regulatory T cells, etc.) (Figure 10B). Subsequently, we constructed butterfly plots to explore the relationship between IMI levels and immune cell infiltration as well as immune function. Apart from APC co-stimulation and HLA, the other 11 immune-related pathways also showed significant correlation with IMI (Figure 10C). Moreover, except for Tfh, Neutrophils, and iDCs, most immune cells exhibited a significant correlation with IMI (Figure 10C). Then, to further validate, we utilized the ESTIMATE algorithm to investigate tumor immune infiltration. We found that the ESTIMATE score and Immune score of the high-IMI group were significantly higher than those of the low-IMI group, while the corresponding Tumor Purity score was significantly lower than that of the low-IMI group (Figure 10D–G). These findings collectively demonstrate increased immune activity within the high-IMI group.

Immune checkpoint inhibitors (ICIs) represent a promising class of cancer therapeutics aimed at enhancing patients’ immune function to counteract tumor cell proliferation [37]. Given the reported correlation between immune checkpoint gene (ICG) expression and the clinical effectiveness of ICIs, we conducted comparative analyses of ICGs in the high-IMI group and the low-IMI group. Our results unveiled substantial disparities in the expression of various ICGs between these IMI groups, highlighting distinct immunological profiles and effects of immunotherapy (Figure 10H). Then we looked into the relationship between two pivotal immune checkpoint genes, *PDCD1* and *CTLA4*, and IMI levels [38,39]. This analysis revealed a significant association, demonstrating that elevated IMI was closely linked to increased expression of these key immune checkpoints (Figure 10I,J).

The discovery further corroborated the conclusion of potential differences in ICI response between the two IMI groups. Additionally, an IPS difference analysis indicated that patients with high IMI receiving combined CTLA4 and PD-1/PD-L1/PD-L2 therapy (*p* = 0.011) might achieve more favorable outcomes compared to individuals with lower IMI (Figure 10K). These findings are in line with earlier studies that have reported positive therapeutic outcomes with combined Nivolumab (PD1 inhibitor) and Ipilimumab (CTLA4 inhibitor) treatment, which has received FDA approval as a first-line treatment for medium- to high-risk advanced RCC [40]. Thus, the outcomes demonstrated the potential of the prognostic signature to indicate the status of immune infiltration and anticipate the reaction to immunotherapy. Since we lacked data on ccRCC patients receiving immune therapy, we acquired the IMvigor210 dataset, comprising 348 patients undergoing anti-PD-1 treatment, as an external cohort. This dataset was used to validate our prognostic IMI characteristics and provide indirect evidence for predicting the efficacy of immune therapy in ccRCC patients. The analysis indicated that patients achieving complete response (CR) or partial response (PR) exhibited increased IMI levels (Figure 10L). Conversely, a larger proportion of patients in the low-IMI group showed disease progression (PD) or stable disease (SD) (Figure 10M). These findings suggest that individuals with elevated IMI levels are more likely to benefit from ICI treatment.

### 3.10. Assessing the Predictive Value of Prognostic Signature in Antineoplastic Drug Sensitivity

**Figure 11:**
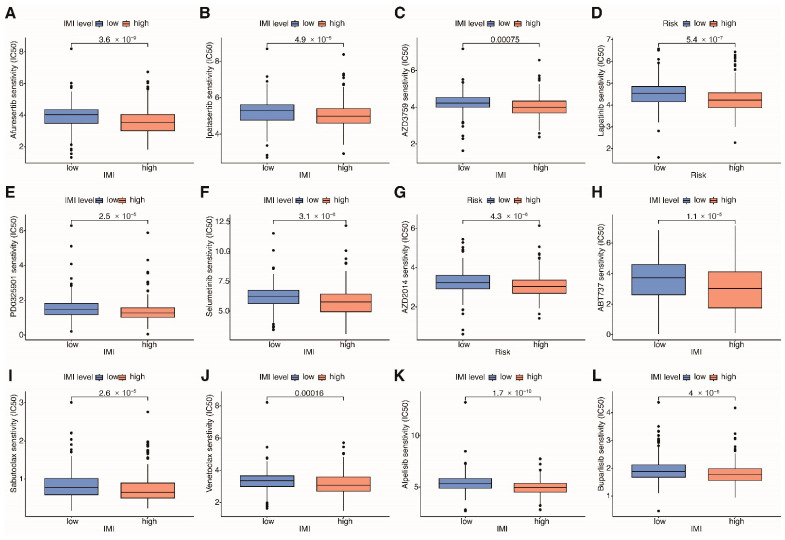
Antineoplastic drug sensitivity prediction. (**A**–**L**) Twelve drugs with higher sensitivity in the low-IMI group.

Apart from ICI therapy, we also aim to predict the sensitivity of commonly used anticancer drugs in renal cancer patients, which is assessed by analyzing the IC50 values. Patients in the high-IMI group with poor prognosis show significantly lower IC50 values for a variety of drugs, indicating increased sensitivity. These drugs include the pan-AKT pathway inhibitors Afuresertib and Ipatasertib, EGFR receptor inhibitors AZD3759 and Lapatinib, MEK inhibitors PD0325901 and Selumetinib, the mTOR inhibitor AZD2014, Bcl inhibitors ABT737, Sabutocla, and Venetoclax, as well as PI3K pathway inhibitors Alpelisib and Buparlisib, etc. (Figure 11A–L).

### 3.11. Identification of Expression Trends of Nine IMRGs

**Figure 12:**
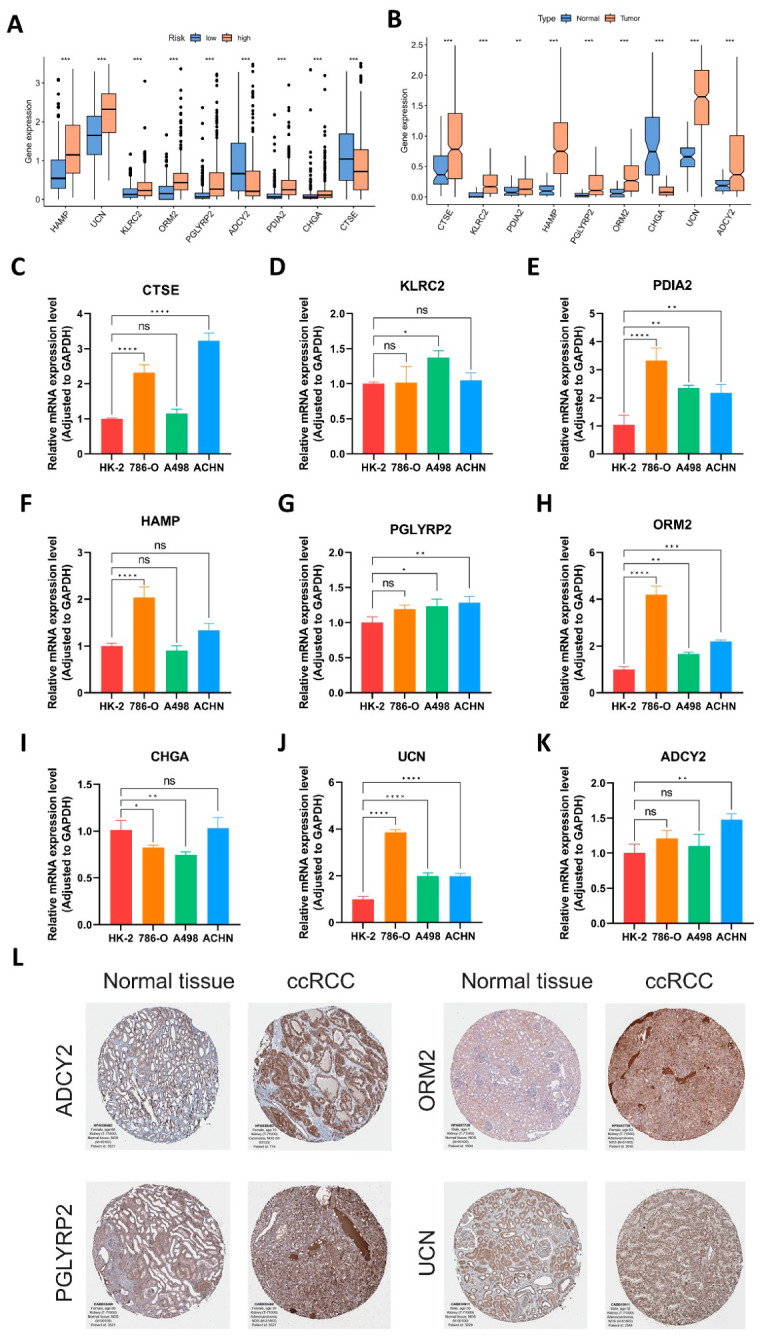
Identification of expression trends of nine IMRGs. (**A**) Differences in signature gene expression between high and low IMI groups in the TCGA database. ns, not significant; * *p* < 0.05; ** *p* < 0.01; *** *p* < 0.001; **** *p* < 0.0001. (**B**) Differences in signature gene expression between normal kidney tissue samples and ccRCC samples in the TCGA database. (**C**–**K**) The relative expression levels of signature genes between three ccRCC cell lines (786-O, A498, ACHN) and normal renal tubular epithelial cells, HK2. (**L**) The IHC images compared the expression levels of four signature genes between normal renal tissue samples and ccRCC samples in the HPA database (https://www.proteinatlas.org, accessed on 1 January 2024).

We examined the expression differences in IMRGs between the high- and low-IMI groups in the TCGA database. Our findings revealed that in the high-IMI group, seven risk genes exhibited higher expression, while the expression of two protective genes was lower, aligning with our expectations (Figure 12A). Subsequently, we compared the expression levels of nine IMRGs between normal kidney tissue samples and ccRCC samples in the TCGA database, and observed significant differences in all nine genes (Figure 12B). However, the two protective genes, *CTSE* and *ADCY2*, did not exhibit low expression levels in tumor tissue; conversely, the risk gene *CHGA* demonstrated low expression levels in tumor tissue. To assess the accuracy and universality of these differential expressions, we conducted validation experiments in a renal tubular epithelial cell line, HK2, and three ccRCC cell lines: 786-O, A498, and ACHN. Through qRT-PCR experiments on these four cell lines, we validated the relative expression levels of nine IMRGs in three ccRCC cells and HK2 cells, and the results were consistent with those from the TCGA database (Figure 12C–K). Although not all nine gene expressions of IMRGs in each ccRCC cell line showed statistical differences from those in HK2 cells, at least one cell line met the predictions of the TCGA database. Furthermore, we validated gene expression via immunohistochemistry (IHC) using the HPA database. Only four genes were available in the database, and the IHC results were consistent with those from TCGA and our experimental findings (Figure 12L).

### 3.12. Verification of UCN Promoting Proliferation, Migration, and Invasion of ccRCC

**Figure 13:**
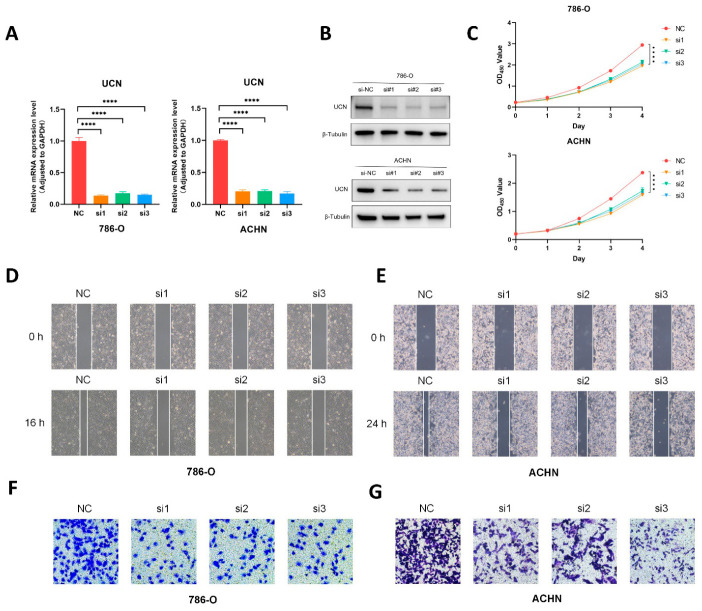
Verification of *UCN* promoting proliferation, migration, and invasion of ccRCC. (**A**) Knockdown of the *UCN* gene in 786-O and ACHN cells, relative mRNA levels in the negative control (NC) group and three siRNA knockdown groups, respectively. **** *p* < 0.0001 (**B**) The knockdown effect of three siRNAs on the *UCN* gene at the protein level in two cell lines. The uncropped blots are shown in Supplementary Figure S4A,B. (**C**) The proliferation curves of CCK8 in the control group and the knockdown groups of the two cell lines. Any siRNA group has significant statistical differences from the NC group. (**D**,**E**) Wound-healing assays in control and knockdown groups of the two cell lines. (**F**,**G**) Transwell invasion assays in control and knockdown groups of the two cell lines.

Our signature indicated that *UCN* had the greatest impact on predicting poor prognosis in ccRCC patients, meaning that *UCN* had the highest coefficient in the signature, so we conducted cell experiments to verify the role of this gene. In previous experiments, we found that *UCN* was significantly overexpressed in the ccRCC cell lines 786-O and ACHN (Figure 12J). Therefore, we knocked down *UCN* in these two cell lines using three independent siRNAs and assessed the knockdown efficiency using qRT-PCR and Western blot (Figure 13A,B). We measured the proliferation levels of ccRCC cells using the CCK8 assay and found that knocking down the *UCN* gene significantly reduced the proliferation levels of the 786-O and ACHN cell lines (Figure 13C). Next, we assessed the migration and invasion abilities of the cells using wound-healing and Transwell invasion assays. The results showed that knocking down the *UCN* gene also inhibited the migration and invasion abilities of 786-O and ACHN (Figure 13D–G).

### 3.13. UCN Regulates the Immunometabolic Microenvironment and Promotes ccRCC Progression

**Figure 14:**
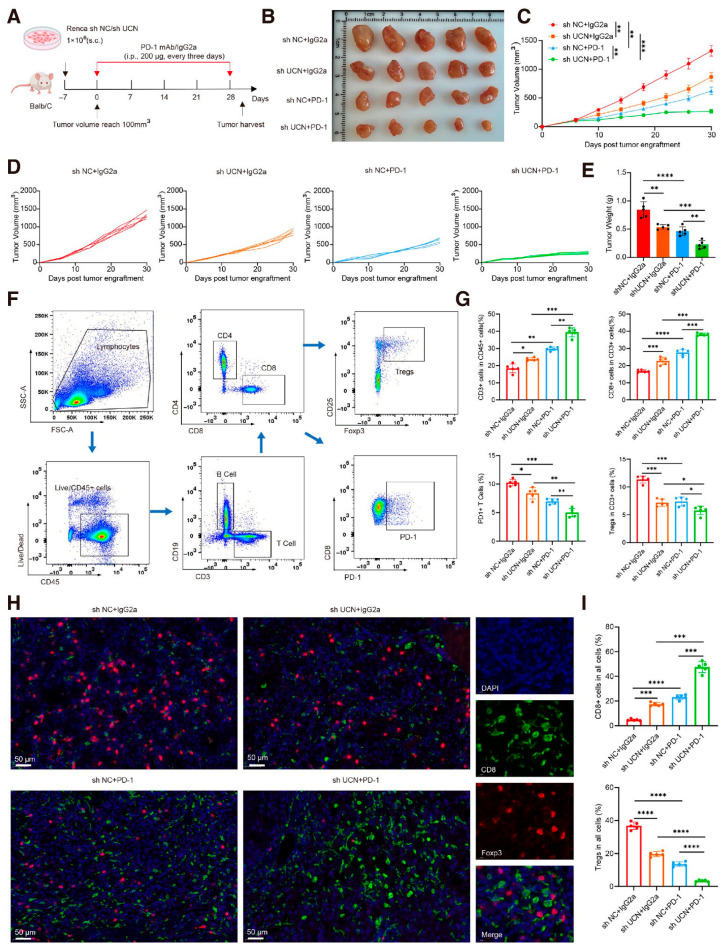
*UCN* regulates the immune microenvironment and promotes ccRCC progression. (**A**) Schematic illustration of the mouse xenograft tumor model experimental design. (**B**–**E**) Tumor growth analyses demonstrate reduced tumor volume and weight across different experimental groups, with notable suppression in sh*UCN*+IgG2a and sh*UCN*+PD-1 groups. (**F**) Gating strategy for tumor-infiltrating lymphocytes. Representative flow plots showing the identification of Live/CD45+ cells, T cells (CD3+), CD4+ and CD8+ subsets, as well as Tregs and PD-1+ cells. (**G**) Flow cytometry analysis unveils substantial alterations in immune cell subsets in the tumor immune microenvironment. (**H**,**I**) Representative mIHC staining of tumors (green: CD8, red: Foxp3, blue: DAPI; scale bar, 50 μm.) (**I**) The column diagram showing the counts of spots with CD8+ T cells and Tregs in tumor slides. Data presented as Mean ± SEM. One-way ANOVA was used in (**E**,**G**,**I**). * *p* < 0.05; ** *p* < 0.01; *** *p* < 0.001; **** *p* < 0.0001.

To further validate *UCN*’s biological function in tumor growth, we constructed a mouse xenograft tumor model (Figure 14A). The in vivo experimental results were highly consistent with the in vitro research findings. We established four experimental groups: shNC+IgG2a (control group), shNC+PD-1, sh*UCN*+IgG2a, and sh*UCN*+PD-1. Compared to the control group, the *UCN* knockdown group (sh*UCN*+IgG2a) showed significantly inhibited tumor growth, with markedly reduced tumor volume and weight (Figure 14B–E, Supplementary Figure S3D). During the observation period, the tumor volume growth rate of the *UCN* knockdown group was significantly slowed. Notably, the *UCN* knockdown group combined with PD-1 inhibition (sh*UCN*+PD-1) demonstrated more significant tumor growth suppression, with further reduced tumor volume and weight compared to the group with *UCN* knockdown alone. To deeply explore *UCN*’s impact on the tumor immune microenvironment, we performed a detailed analysis of immune cell subsets in tumor tissues using flow cytometry (Figure 14F,G). The results showed significant changes in immune cell distribution in the *UCN* knockdown group compared to the control group. Specifically, the proportions of CD3+ T cells and CD8+ T cells were significantly increased. The proportions of PD-1+ T cells and Tregs were significantly decreased. This finding suggests that *UCN* may influence tumor progression by regulating immune cell infiltration. Notably, the *UCN* knockdown group combined with PD-1 inhibition (sh*UCN*+PD-1) showed further enhanced immune cell activity, implying a potential synergistic effect between *UCN* and PD-1. Multiplex immunohistochemical images clearly demonstrated that CD8+ T cell infiltration was significantly higher and Treg infiltration was significantly lower in the *UCN* knockdown group compared to the control group (Figure 14H,I), further confirming our previous findings. Previous literature has described *UCN*s as key regulators of energy homeostasis and glucose metabolism, playing a critical bridging role between nutritional status and metabolic responses [41,42,43]. In the specific pathological context of ccRCC, we found that *UCN* exerts a metabolic driving effect by upregulating key glycolytic enzymes (Supplementary Figure S3E). Our experimental results are consistent with the established role of the *UCN* family in metabolic regulation. This finding aligns with the broad recognition of *UCN*s as potent modulators of glucose-related pathways. In conclusion, *UCN* demonstrates multiple regulatory functions in ccRCC development, providing an important theoretical basis for *UCN* as a potential therapeutic target.

## 4. Discussion

Notably, RCC constitutes the third most prevalent cancer of the urinary system and carries a significant mortality rate. Despite ongoing efforts to develop effective treatments for ccRCC, it remains a significant challenge in the field of oncology, and further studies are required to fully understand this complex disease. The identification of biomarkers that are definitive in predicting prognosis and steering RCC treatment in a clinical environment is still insufficient.

In recent years, cancer metabolism has garnered considerable attention from researchers due to its dysregulation in tumors. The reprogramming of cellular metabolism in ccRCC is mainly attributed to oncogenic mutations, thereby identifying it as a metabolic ailment [44]. Metabolic reprogramming in tumor cells facilitates the synthesis of cellular components, involving DNA, membrane structures, and molecules that modulate the energy balance of tumors. Otto Heinrich Warburg’s observation that most cancer cells generate energy through aerobic glycolysis has particular relevance to ccRCC since this phenomenon is more pronounced in this form of cancer compared to normal tissues [45,46]. Additionally, abnormal lipid metabolism is a prominent feature of ccRCC, with substantial lipid accumulation observed in ccRCC cells. Compared to normal tissues, ccRCC cells exhibited a substantial elevation in the concentrations of cholesterol, triglycerides, and cholesterol esters [47]. The anomalous production of fatty acids and cholesterol provides energy substrates and membrane components that are necessary for the proliferation of rapidly growing tumor cells. This phenomenon enables tumor cells to adapt continually to various microenvironmental conditions that favor their growth [48].

There is mounting evidence of a strong association between immune cell responses and metabolic reprogramming. Recent studies suggest that certain tumor cells enhance fatty acid uptake, leading to the suppression of CD8+ T cells [49]. Moreover, researchers have found that integrating an anti-CTLA4 antibody with an inhibitor of fatty acid transporter 2 has been found to be an effective approach in halting tumor progression [50]. Additionally, the oncometabolite d-2-hydroxyglutarate (d-2HG) targets glycolytic enzyme lactate dehydrogenase, and metabolic pathway alterations and an immune CD8+ T cell signature characterized by suppressed cytotoxicity and compromised interferon-γ signaling are induced by d-2HG [13]. These outcomes demonstrated that environmental factors within the tumor, as well as metabolic adaptations in cells and the presence of immune cell infiltration, could impact treatment responsiveness. The growing field of immunometabolism is associated with promising possibilities for utilizing immunotherapy as a therapeutic approach for cancer.

Our study conducted a thorough investigation of IMRGs related to prognosis using clinical and sequencing data sourced from TCGA-KIRC, leading to the molecular classification of ccRCC patients. The results of molecular classification showed significant differences in prognosis and other aspects between the two subtypes, and we developed a prognostic signature based on the DEGs of the two subtypes. This signature performed well in both internal and external validation. Furthermore, we compared our signature with ten published models that focused solely on immunity or metabolism, and our results demonstrated that our signature outperformed all of them. Significant differences were observed in survival and clinical characteristics between high- and low-IMI groups. Moreover, significant variations were also observed in TME, immune infiltrating cells, and immune checkpoints. Furthermore, evaluating the response to ICIs through the IPS scores demonstrated the superiority of *PD1* and *CTLA4* combined immunotherapy. Through the integration of clinical parameters identified via univariate and multivariate Cox regression analyses with IMRGs, a nomogram was developed to provide a more precise prediction of the prognosis and survival risk of patients with ccRCC. The utilization of this combined approach has shown promising results in enhancing the accuracy of prognostic prediction for ccRCC patients. We functionally characterized *UCN*, the key risk gene in our signature. *UCN* is a neuropeptide related to the stress response. Beyond its known roles, our work establishes its oncogenic function in ccRCC. *UCN* promoted tumor cell aggressiveness in vitro and in vivo. Crucially, *UCN* shaped an immunosuppressive TME by limiting CD8+ T cell infiltration and enriching Tregs. The synergistic antitumor effect observed between *UCN* knockdown and PD-1 blockade provides a strong rationale for targeting *UCN* to overcome immune resistance. The precise mechanism by which *UCN* exerts these immunomodulatory effects warrants further investigation but may involve direct signaling on immune cells or indirect effects via cytokine/chemokine regulation.

Our study acknowledges certain limitations, which open several avenues for future research. Primarily, large-scale, multi-center prospective clinical trials are warranted to further validate the predictive power and clinical utility of the IMI signature in diverse real-world settings. Additionally, further in-depth mechanistic studies are essential to fully elucidate the specific receptors and downstream signaling pathways through which the *UCN*-driven immunometabolic axis modulates the interaction between cancer cells and CD8+ T cells. Lastly, integrating multi-omics data into future models could enhance their clinical utility for non-invasive monitoring and precision oncology. Such advancements will provide a more robust theoretical basis for overcoming immunotherapy resistance in ccRCC.

## 5. Conclusions

In summary, by integrating immunity and metabolism-related genes, we developed a novel prognostic signature that accurately predicts risk and immunotherapy response in ccRCC, offering significant potential to improve clinical management. A personalized nomogram was established as a practical tool for clinical decision-making. Functional validation identified *UCN*, the gene with the highest risk coefficient, as a key promoter of ccRCC progression: it significantly enhanced tumor cell proliferation, migration, and invasion, and reshaped the immune microenvironment by regulating CD8+ T cell and Treg infiltration.

## Data Availability

The RNA-seq and clinical data of ccRCC samples were accessed from the TCGA database (https://portal.gdc.cancer.gov/, accessed on 1 January 2024). The IPS was obtained from the TCIA database (https://tcia.at/home, accessed on 1 January 2024). The Array Express database with identifier E-MTAB-1980 was obtained from https://www.ebi.ac.uk/arrayexpress, accessed on 1 January 2024. Additionally, a comprehensive data package, licensed under Creative Commons 3.0, is available for download at http://research-pub.gene.com/IMvigor210CoreBiologies (accessed on 1 January 2024). All data and materials used in this study are available from the corresponding author upon reasonable request.

## Supplementary Materials

The following supporting information can be downloaded at https://www.mdpi.com/article/10.3390/cancers18091373/s1: Supplementary Figure S1: Optimal rank value determination for patient clustering. (A) The NMF divided patients into 2-10 clusters, and the results showed that the classification into 2 clusters was the best. (B) NMF algorithm characteristics. Supplementary Figure S2: Details of genomic alteration landscapes between the two molecular subtypes. (A) Variant classification of the cluster 1. (B) Variant type of the cluster 1. (C) SNV class of the cluster 1. (D) Variant frequency for each sample in cluster 1. (E) Variant classification summary of the cluster 1. (F) The proportion of each variant classification in the top 10 mutated genes from the cluster 1. (G) Variant classification of the cluster 2. (H) Variant type of the cluster 2. (I) SNV class of the cluster 2. (J) Variant frequency for each sample in cluster 2. (K) Variant classification summary of the cluster 2. (L) The proportion of each variant classification in the top 10 mutated genes from the cluster 2. Supplementary Figure S3: IMRG signature benchmarking and UCN-related metabolic profiling in ccRCC. (A) The prognostic signature based on IMRG can distinguish between ccRCC patients with good and poor prognosis. (B,C) Other previously published signatures can also distinguish ccRCC patients. (D) Two types of shRNAs showed knockout effects on the UCN gene at the protein level (we chose sh # 1 with better efficacy for further research). The uncut imprint is shown in Supplementary Figure S4C. (E) qRT-PCR analysis of glycolysis-related genes (GLUT1, HK2, LDHA, and PKM2) following UCN knockdown. * *p* < 0.05, ** *p* < 0.01, *** *p* < 0.001. Supplementary Figure S4: Original uncropped images for all Western blots. (A, B) Serving as supplemental data for Figure 13B in the main text; (C) Serving as supplemental data for Supplementary Figure S3D. Supplementary Table S1: Immune function genes that applied in the analysis. Supplementary Table S2: Metabolic function genes that applied in the analysis. Supplementary Table S3: Differentially expressed gene analysis of IMRGs. Supplementary Table S4: Prognostical IMRGs that applied in the analysis. Supplementary Table S5: The primer sequences for gene expression validation. Supplementary Table S6: The sequences of siRNAs for UCN knockdown.

## Abbreviations

The following abbreviations are used in this manuscript:

AUC	Area under the curve
ccRCC	Clear cell renal cell carcinoma
ICI	Immune checkpoint inhibitor
IMI	Immune Metabolic Index
IMRG	Immune- and metabolism-related gene
IPS	Immunophenoscore
NMF	Non-negative matrix factorization
DEGs	Differentially expressed genes
OS	Overall survival
PFS	Progression-free survival
RCC	Renal cell carcinoma
ROC	Receiver-operating characteristic
RS	Risk score
TCGA	The Cancer Genome Atlas
TKI	Tyrosine kinase inhibitor
TMB	Tumor mutation burden
TME	Tumor microenvironment
