# Supplementary figures and images for: An Integrated Immunometabolic Signature Predicts Prognosis and Immunotherapy Response in ccRCC and Identifies UCN-Mediated Immune Evasion as a Therapeutic Vulnerability: Evidence from In Vitro and In Vivo Studies
Source: Cancers (Basel). 2026 Apr 25;18(9):1373. doi: 10.3390/cancers18091373 (PMC13163016; doi:10.3390/cancers18091373)

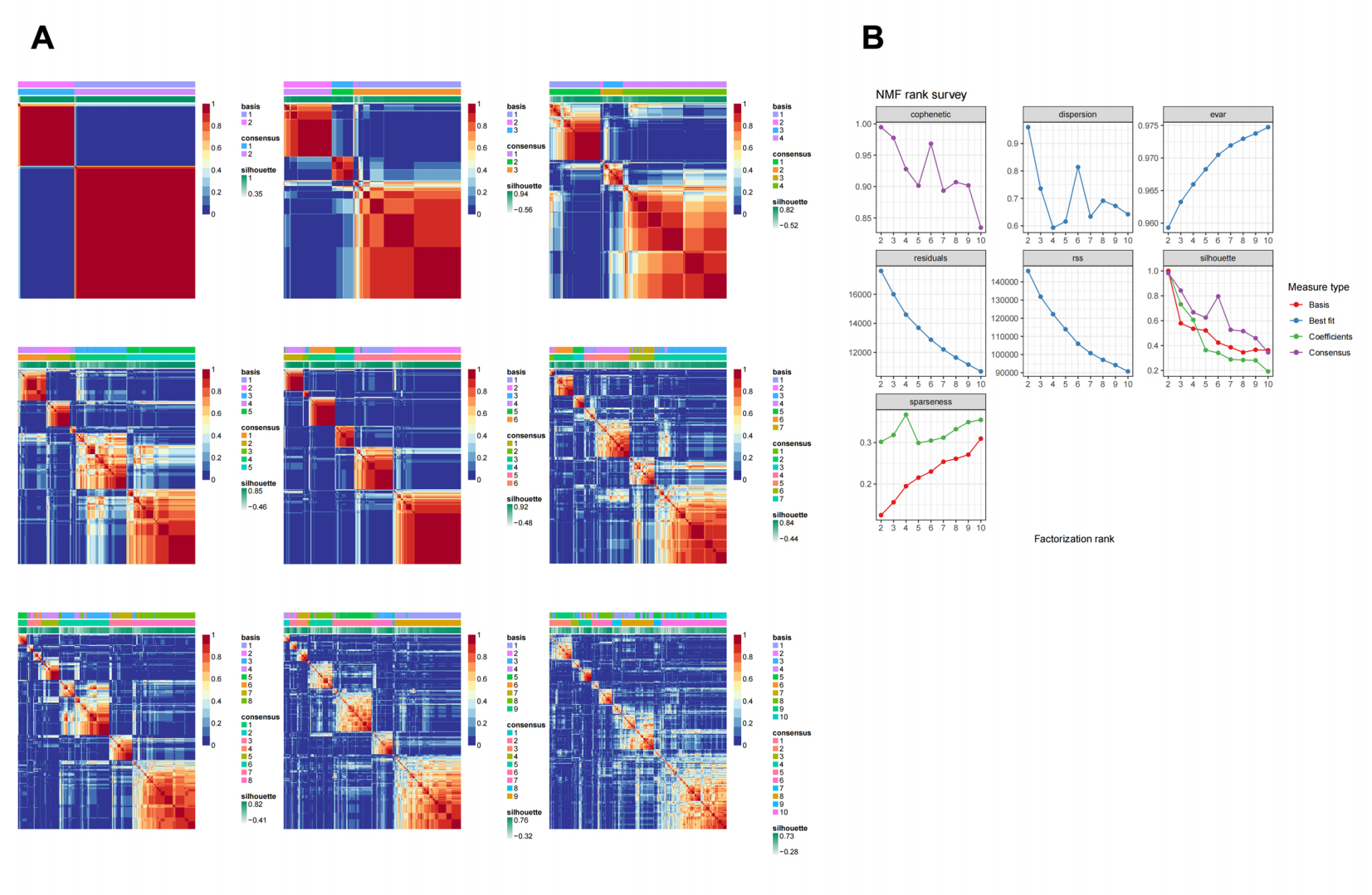

Supplement: Supplementary file 1 [file cancers-18-01373-s001.zip › Supplementary Figure S1.tif]

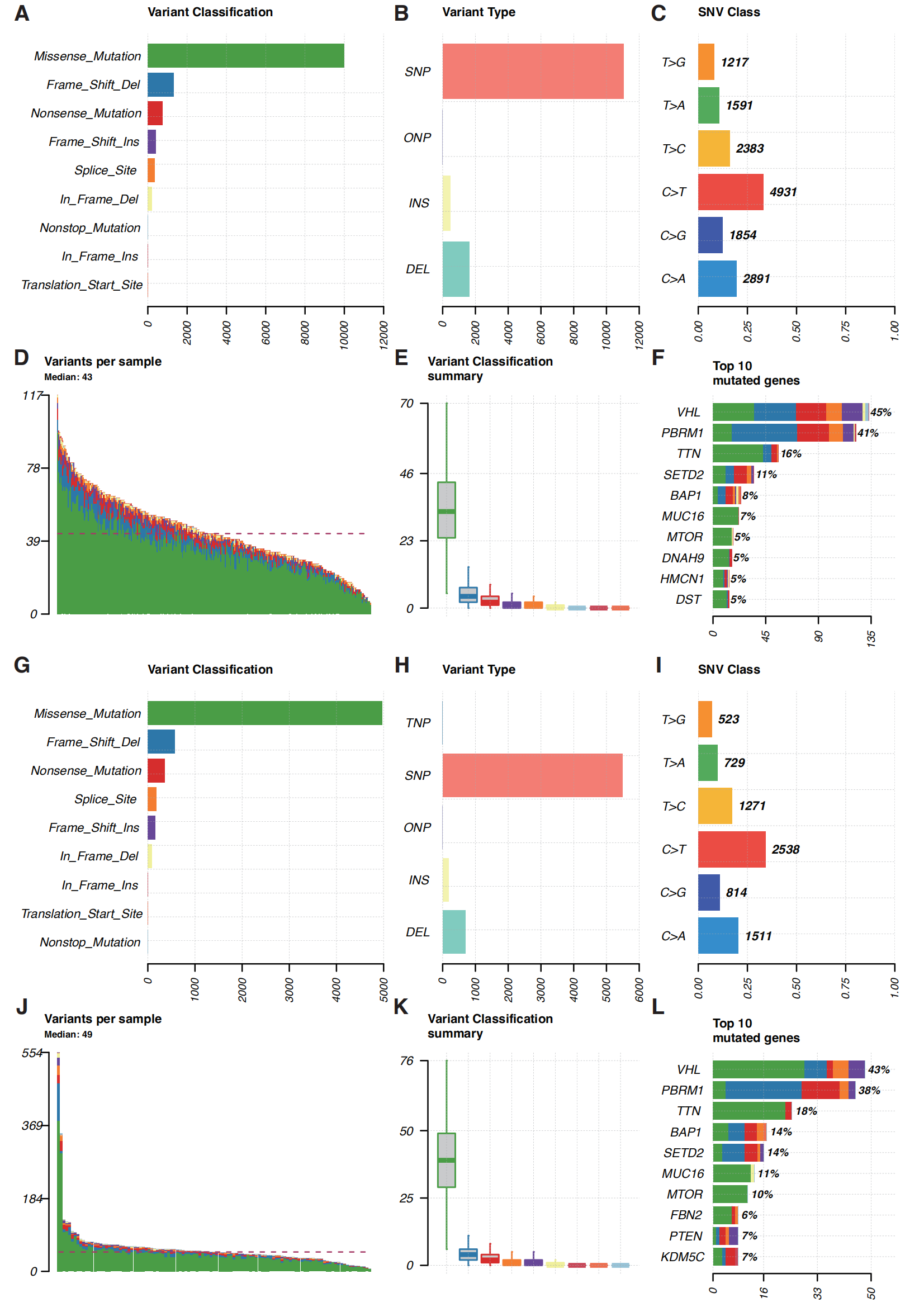

Supplement: Supplementary file 1 [file cancers-18-01373-s001.zip › Supplementary Figure S2.tif]

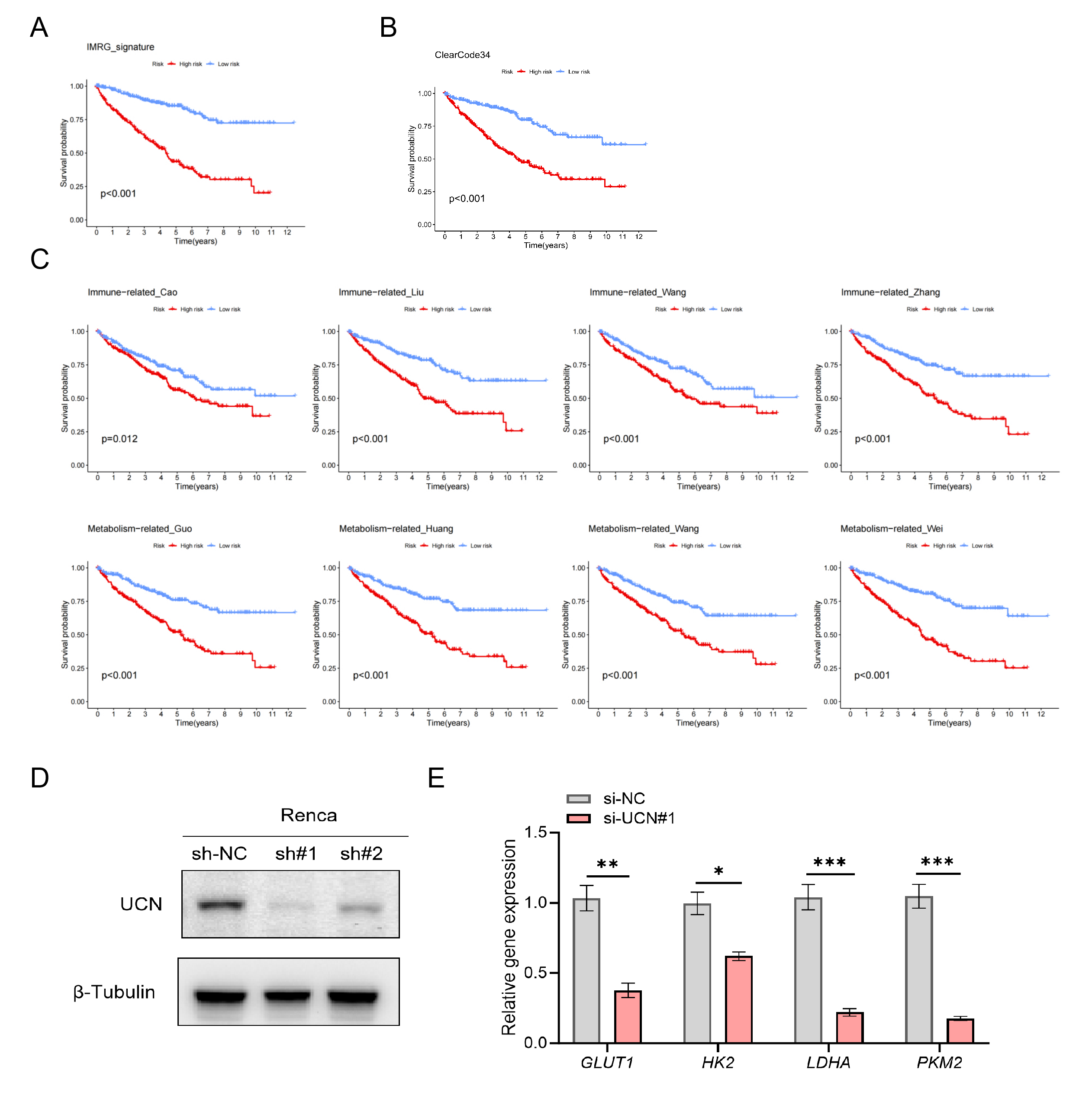

Supplement: Supplementary file 1 [file cancers-18-01373-s001.zip › Supplementary Figure S3.tif]

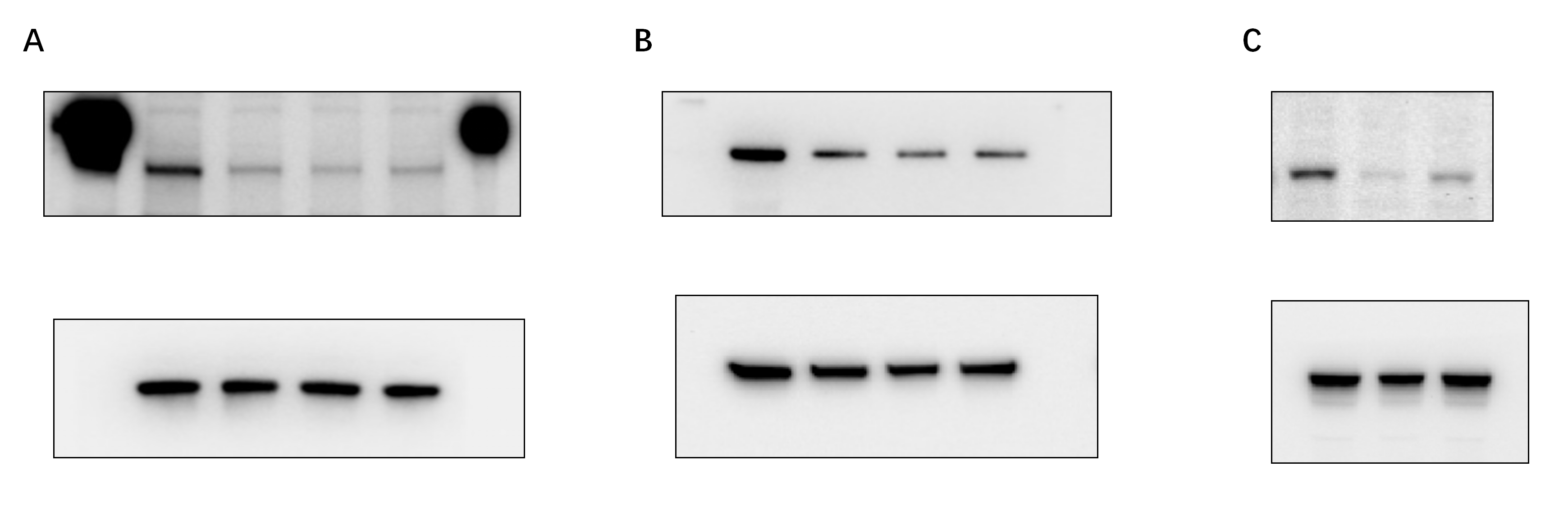

Supplement: Supplementary file 1 [file cancers-18-01373-s001.zip › Supplementary Figure S4.tif]
